# Global research landscape and thematic transitions in pyruvate kinase deficiency: a decadal bibliometric analysis (2015–2025)

**DOI:** 10.3389/fmed.2026.1865929

**Published:** 2026-06-24

**Authors:** Wanyi Zhao, Yang Wang, Na Gao, Wenzheng Yu, Zengyan Liu

**Affiliations:** Department of Hematology, Binzhou Medical University Hospital, Binzhou, Shandong, China

**Keywords:** bibliometrics, multi-database validation, pyruvate kinase deficiency, rare disease bibliometrics, research hotspots, visualization analysis

## Abstract

**Background:**

Pyruvate kinase deficiency (PKD) is the most common cause of congenital non-spherocytic hemolytic anemia. This study systematically maps the scholarly output and evolving research trends in PKD over the past decade (2015–2025) to identify core contributors and track thematic trajectories.

**Methods:**

Bibliographic records were retrieved from the Web of Science Core Collection (WoSCC) and analyzed quantitatively using CiteSpace, VOSviewer, and R-bibliometrix. To control for potential selection bias, a parallel supplementary search was conducted in PubMed and Scopus using identical query strings. PubMed yielded 15 unique non-overlapping eligible records, whereas Scopus returned 0. Given metadata disparities and small sample limits, these records were dedicated exclusively to exploratory qualitative thematic concordance verification rather than statistical network synthesis.

**Results:**

The primary WoSCC network isolated 90 core publications derived from 649 initial records after rigorous screening. Multi-database cross-validation confirmed a stable thematic alignment. Manual textual extraction of the 15 external PubMed records displayed precise semantic resonance with the core dataset’s clusters, tracking a clear focus transition from baseline genotype mapping to targeted pyruvate kinase, R-type (PKR) activators (specifically mitapivat) and preclinical gene-editing interventions, without introducing divergent thematic anomalies.

**Conclusion:**

The analytical framework indicates that the PKD research domain is characterized by an evolving therapeutic focus, progressing from foundational descriptive epidemiology and supportive care toward mechanism-based, targeted interventions. Current empirical evidence highlights PKR allosteric activation as the most clinically developed cluster, while gene therapy represents an active, early-stage investigational frontier.

## Introduction

Pyruvate kinase deficiency (PKD), arising from homozygous or compound heterozygous mutations in the *PKLR* gene, manifests clinically as chronic non-spherocytic hemolytic anemia. This condition is frequently associated with secondary complications, including systemic iron overload, pulmonary hypertension, and extramedullary hematopoiesis (1). While Pyruvate kinase (PK) enzymatic activity assays remain the standard diagnostic approach, their reliance on concomitant reticulocyte counts can compromise baseline reliability in atypical phenotypes, occasionally resulting in diagnostic delays (2, 3). Consequently, the integration of enzymatic screening with molecular DNA analysis has emerged as a verified strategy to optimize diagnostic precision (4).

As the leading cause of congenital non-spherocytic hemolytic anemia (CNSHA) worldwide, PKD carries a global prevalence estimated between 1/100,000 and 1/300,000 (5). Retrospective epidemiological parameters indicate that PKD cohorts exhibit a truncated median overall survival compared to healthy control groups (10.9 vs. 17.1 years), which corresponds to a quantified 2.3-fold increase in mortality risk (6). Historically, clinical management has been restricted to supportive and symptomatic interventions—chiefly regular blood transfusions, splenectomy, and iron chelation therapy. However, the therapeutic landscape has expanded with the clinical development of the small-molecule PKR activator mitapivat (7). Although allogeneic hematopoietic stem cell transplantation offers a definitive biological cure, its general clinical utility remains constrained by donor availability and transplant-related morbidity (8). Furthermore, while splenectomy effectively increases baseline hemoglobin levels and alleviates transfusion dependency, these clinical outcomes must be continuously weighed against documented postoperative risks (9).

Against this evolving clinical backdrop, bibliometric analysis provides an empirical, data-driven methodology to systematically evaluate research trajectories and map structural shifts within the literature. Rather than replicating narrative summaries, this study applies computational scientometric tools to construct a macro-level structural framework of the PKD literature from 2015 to 2025, aiming to offer objective parameters to guide prospective clinical investigations and resource allocation.

## Materials and methods

### Data extraction and standardized query architecture

A systematic literature search was conducted covering the temporal window from July 2015 to July 2025 using the Web of Science Core Collection (WoSCC) as the primary data repository. The retrieval was specifically restricted to the Science Citation Index Expanded (SCI-EXPANDED) and the Social Sciences Citation Index (SSCI). To maintain strict methodological symmetry and ensure cross-database comparability, an identical standardized free-text search string was executed across the primary and validation repositories. The exact search syntax and queries were defined as follows:

(1) WoSCC: TS = (“Pyruvate Kinase Deficiency”);
(2) PubMed: “Pyruvate Kinase Deficiency”[Title/Abstract];
(3) Scopus: TITLE-ABS-KEY (“Pyruvate Kinase Deficiency”).

No manual Boolean OR operators or truncation symbols (e.g., asterisks) were utilized for synonym expansion at the primary retrieval stage. This conservative strategy was intentionally adopted because “Pyruvate Kinase Deficiency” represents the definitive medical and clinical standard nomenclature for this specific rare condition. Broader terms, such as CNSHA, encompass a highly heterogeneous spectrum of independent disorders (including G6PD deficiency and other distinct enzymopathies); thus, their inclusion would introduce substantial background noise and irrelevant bibliographic nodes, potentially distorting the specificity and structural integrity of the rare-disease network topology. Furthermore, MeSH terms and database-specific controlled vocabularies were deliberately excluded. Because MeSH indexing is exclusive to PubMed/MEDLINE and frequently subject to indexing time lags, its application would systematically bias the retrieval symmetry and introduce cross-platform metadata incompatibility when compared directly with the WoSCC and Scopus indexing frameworks.

To guarantee the transparency and reproducibility of our literature retrieval framework, the exhaustive technical search syntax, specific field tags, and comprehensive filtering workflows across all utilized digital repositories (WoSCC, PubMed, and Scopus) are systematically cataloged in Supplementary Table S1.

**FIGURE 1:**
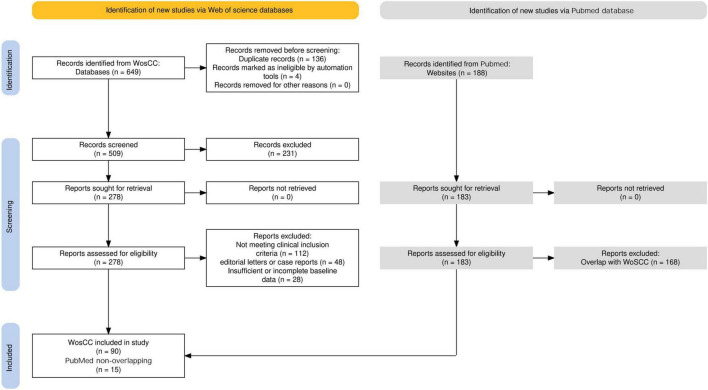
The flow chart of literature screening and data analysis process.

The inclusion criteria were further limited to English-language original articles and review articles. The initial bibliographic retrieval from WoSCC yielded 649 PKD-related records. Following a rigorous multi-stage screening process (modeled via the PRISMA 2020 framework in Figure 1), 114 duplicate records were removed, 365 records were excluded based on title and abstract screening, and 80 records were excluded during full-text eligibility assessment based on predefined clinical criteria. Ultimately, 90 core publications were selected for primary network analysis, comprising 62 original research articles and 28 reviews. Concurrently, the harmonized search in PubMed yielded 188 initial hits, which after identical technological filtering, produced 15 unique, non-overlapping eligible records utilized exclusively for external thematic concordance checks. To ensure absolute empirical traceability and transparency, the detailed bibliographic descriptors (including PMIDs, DOIs, and functional topic tags such as mitapivat or gene therapy) of these 15 validation articles are structurally cataloged in Supplementary Table S2. Meanwhile, the parallel query executed in Scopus yielded 0 additional relevant entries beyond the baseline dataset, reinforcing the capture efficiency of our primary retrieval framework.

### Data analysis

Bibliographic records were retrieved from the WoSCC database in plain text format and subsequently integrated into CiteSpace (V6.4.R1), VOSviewer (V1.6.20), and the R-bibliometrix package for rigorous data standardization and deduplication. Quantitative metrics—including annual publication trajectories, citation impact, and relative contribution shares—were systematically calculated. The temporal analysis window was established from July 2015 to July 2025, employing a one-year time slice for granular observation. In the resulting knowledge maps, node magnitude encodes the frequency of entities (e.g., countries, institutions, or keywords), while inter-node edges characterize the intensity of collaborative or co-occurrence relationships. To optimize network clarity and mitigate redundancy, node filtering was executed via the g-index (*k* = 25), which serves as a robust threshold to selectively retain highly-cited, intellectually significant nodes while effectively filtering out low-impact background noise (10, 11). Concurrently, the Pathfinder and pruning of merged networks algorithms were applied to distill the primary research framework and highlight emerging thematic trends. Figure 1 shows the flow chart of literature screening and data analysis process.

### Exploratory multi-database cross-validation

To systematically account for potential database-specific selection bias, parallel supplementary literature sweeps were conducted across the PubMed and Scopus networks using an identical keyword framework. This control query identified 0 additional records in Scopus and yielded 15 unique, non-overlapping publications within PubMed (Supplementary Table S2) that were absent from the primary WoSCC cohort. Acknowledging the small sample boundary (*n* = 15) and structural metadata disparities that prevent stable mathematical network synthesis, this parallel search was strictly designated as an exploratory qualitative thematic concordance check rather than a confirmatory statistical topology validation. Instead of calculating network overlap metrics or topological convergence ratios, an investigator-led manual extraction of textual data and citation strings was conducted on these 15 records. This process computed the precise keyword-level conceptual intersection rate to verify whether peripheral datasets mirror the core thematic focus transitions established by the primary WoSCC network architecture.

## Results

### Temporal distribution and co-authorship network dynamics

**FIGURE 2:**
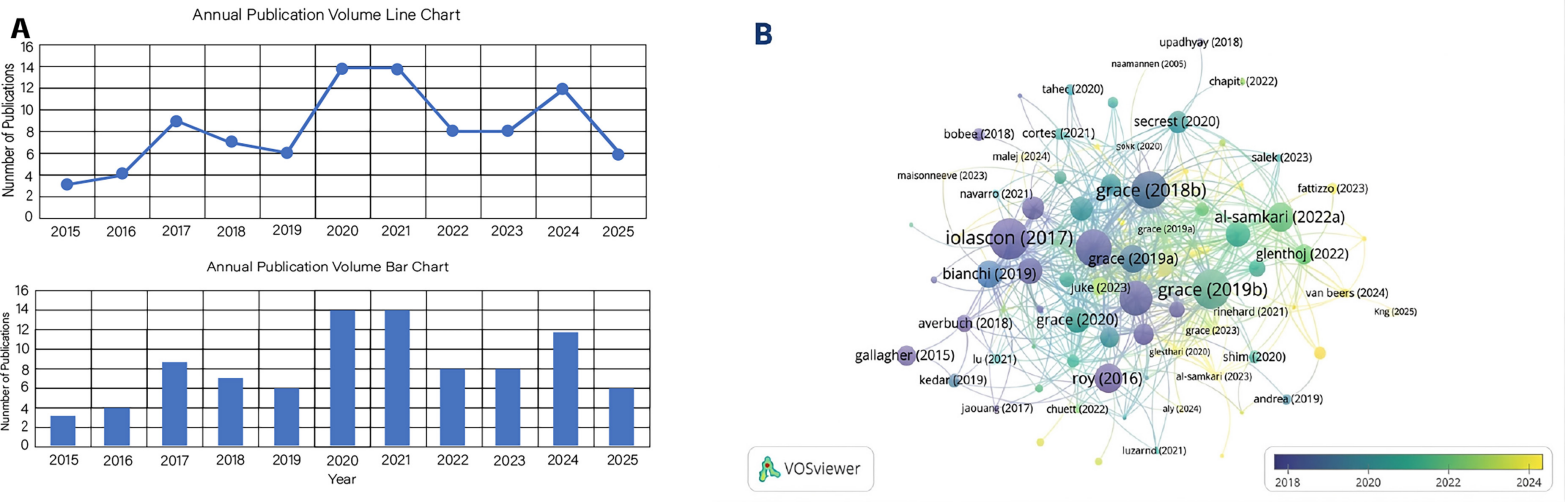
Analysis of annual publication volume. **(A)** Line graph of annual PKD publication volume from 2015 to 2025. **(B)** VOSviewer analysis of PKD publication volume from 2015 to 2025.

As shown in Figure 2A, between 2015 and 2020, the annual output of English-language PKD publications witnessed a steady accrual, culminating in a historical peak in 2021. Although the publication volume experienced a marginal contraction thereafter, research productivity has sustained a robust baseline.

Figure 2B illustrates the structural evolution and temporal dynamics of the PKD literature from 2015 to 2025 via a co-authorship network characterized by active international collaborations. Network analysis isolates a structure anchored by specific high-centrality nodes, where node magnitude mirrors the absolute citation impact of the literature. Foundational studies published in 2016–2017, together with a series of highly cited investigations from 2018 to 2020, occupy the network’s core, serving as primary hubs for knowledge dissemination within the field. Chronologically, the research focus exhibits a distinct thematic transition. Early-stage scholarship (2015–2019) focused primarily on characterizing the natural history and clinical manifestations of PKD. This transitioned into an evolving therapeutic focus between 2020 and 2022, signaling a dense cluster of publications evaluating novel small-molecule PKR activators. Recent nodes (2023–2025) represent the current research frontier, where investigations have moved toward long-term clinical efficacy assessments and early-stage gene therapy models. The dense interconnectivity across the network mathematically indicates structural convergence, tracking the domain’s progression from descriptive epidemiology to targeted clinical interventions.

### Citation analysis of central publication nodes

In terms of citation density within the WoSCC database, Blood, the British Journal of Haematology, and Haematologica emerged as the leading channels of scholarly output, as detailed in Table 1. The clustering of these platforms indicates that the intellectual foundation of PKD research is situated within hematology and erythrocyte metabolic pathology, with expanding integration into clinical therapeutics and pharmaceutical development.

**TABLE 1:** Top 10 most influential journals in PKD research.

Rank	Journal name	Impact factor (2024)	Co-citation frequency (TC)	Publication count	Centrality	Publisher/country
1	Blood	21	82	14	0	American Society of Hematology (USA)
2	British Journal of Haematology	5	78	9	0	Wiley-Blackwell (UK)
3	American Journal of Hematology	10.1	75	11	0.01	Wiley-Blackwell (USA)
4	Haematologica	8.2	65	5	0.03	Ferrata Storti Foundation (Italy)
5	New England Journal of Medicine	96.2	51	1	0.01	Massachusetts Medical Society (USA)
6	Blood Cells, Molecules, and Diseases	2.3	50	5	0.02	Elsevier (USA)
7	European Journal of Haematology	3.1	41	6	0.04	Wiley-Blackwell (UK)
8	Blood Reviews	7	36	0	0.16	Elsevier (UK)
9	Journal of Clinical Investigation	13.3	32	0	0.02	American Society for Clinical Investigation (USA)
10	Pediatric Blood and Cancer	2.4	24	2	0.01	Wiley-Blackwell (USA)

**FIGURE 3:**
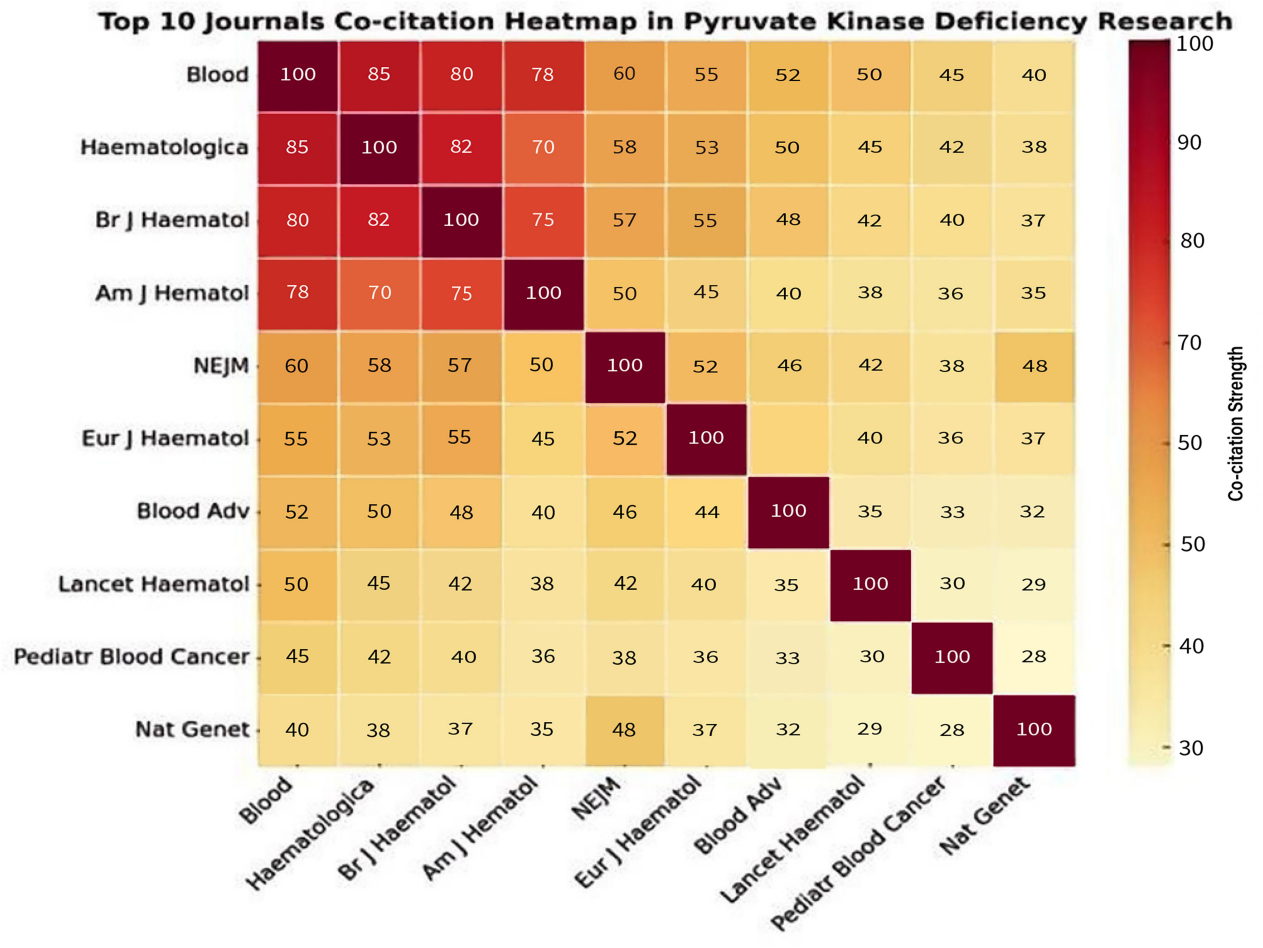
Journal citation intensity thermogram.

Within the citation hierarchy, the high citation volumes of Blood (Co-citation Frequency = 82, Publication Count = 14, Centrality = 0.00) and the British Journal of Haematology (Co-citation Frequency = 78, Publication Count = 9, Centrality = 0.00), complemented by the superior structural centrality of platforms like Haematologica (Centrality = 0.03, Publication Count = 5) and Blood Reviews (Centrality = 0.16, Publication Count = 0), collectively identify these platforms as the central publication nodes of the discipline. This topological distribution is further corroborated by density analysis, which reveals a localized cluster of highly co-cited journals encompassing Blood, Br J Haematol, Am J Hematol, and Haematologica, as illustrated in the density thermogram in Figure 3.

### Collaborative network analysis

#### Distribution of countries and institutions

**FIGURE 4:**
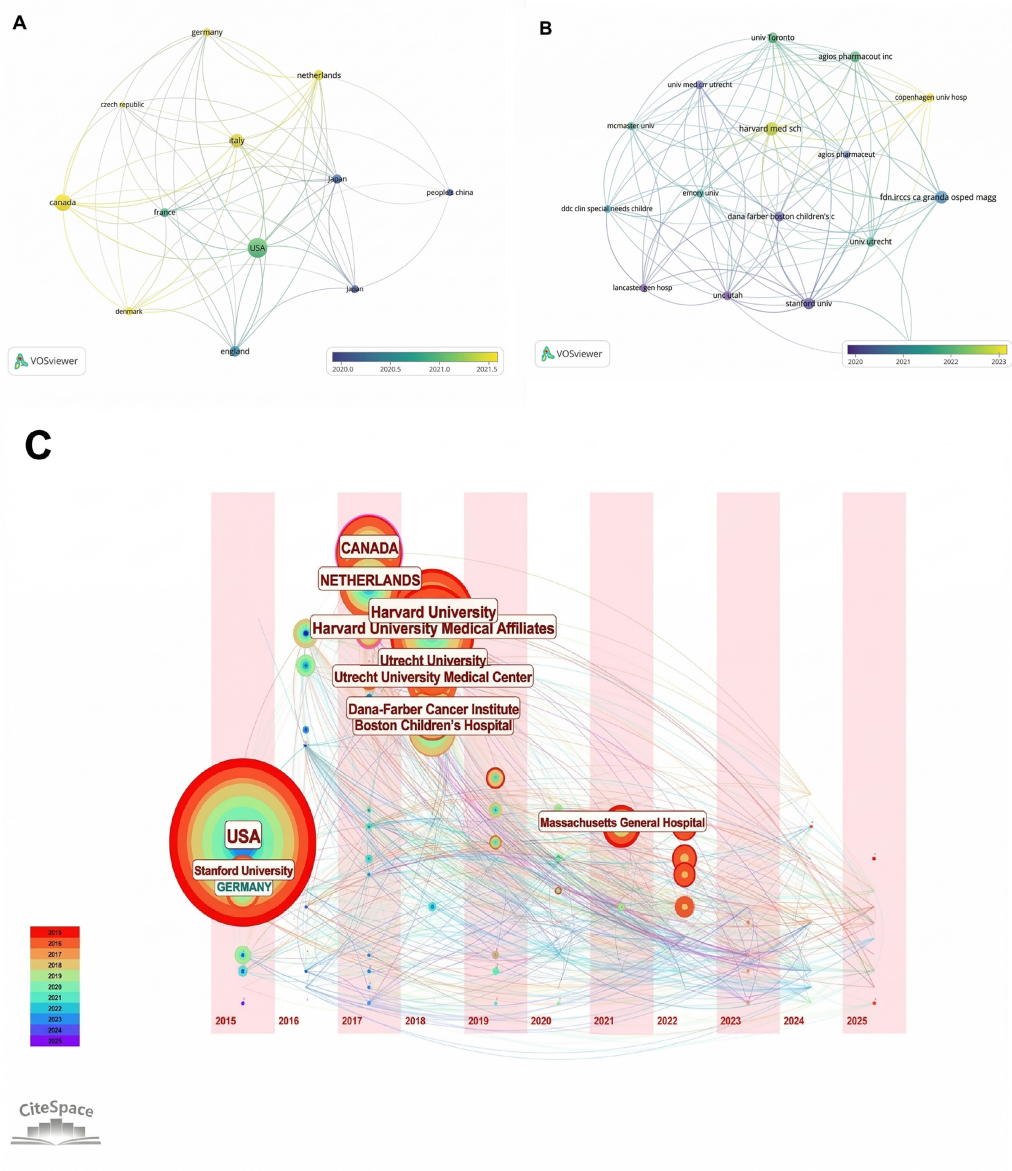
Analysis of countries and institutions. **(A)** VOSviewer analysis chart of national cooperation. **(B)** VOSviewer analysis chart of institutional cooperation. **(C)** Analysis of CiteSpace collaboration among institutions.

The United States represents the highest volume of scholarly output within the PKD research landscape (Publication Count = 47, Centrality = 0.17), exhibiting prominent collaborative links with Italy (Count = 32, Centrality = 0.32) and Canada (Count = 21, Centrality = 0.03). Since 2020, the global cooperative framework has undergone significant topological densification, resulting in a co-authorship network with expanded international collaborations, as shown in Figures 4A,C.

Strategic cross-sector collaborations among prominent institutions—most notably Utrecht University (17 publications, Centrality = 0.01), Agios Pharmaceuticals (17 publications, Centrality = 0.12), and Harvard Medical School (16 publications, Centrality = 0.01)—have intensified, driven by the requirements of clinical translation and novel therapeutic testing. Within the United States, Harvard University and its Medical Affiliates function as highly productive institutional nodes, co-ranking at the top of productivity with 27 publications each (Table 2), commanding central positions in the collaborative network graph shown in Figures 4B,C.

**TABLE 2:** Top 10 most productive countries and institutions in PKD research (2015–2025).

Rank	Top 10 countries	Publication count (n)	Centrality	Top 10 institutions	Publication count (n)	Centrality
1	USA	47	0.17	Harvard University	27	0.01
2	Italy	32	0.32	Harvard University Medical Affiliates	27	0.01
3	Canada	21	0.03	IRCCS Ca Granda Ospedale Maggiore Policlinico	23	0.02
4	Netherlands	20	0.17	Agios Pharmaceuticals	17	0.12
5	England	18	0.25	Utrecht University	17	0.01
6	Spain	13	0.17	Harvard Medical School	16	0.01
7	Germany	13	0.01	Utrecht University Medical Center	16	0.00
8	France	10	0.11	Dana-Farber Cancer Institute	15	0.01
9	Denmark	9	0.01	Boston Children’s Hospital	15	0.01
10	Peoples R China	6	0	Massachusetts General Hospital	13	0.00

#### Author distribution and reference co-citation structures

**FIGURE 5:**
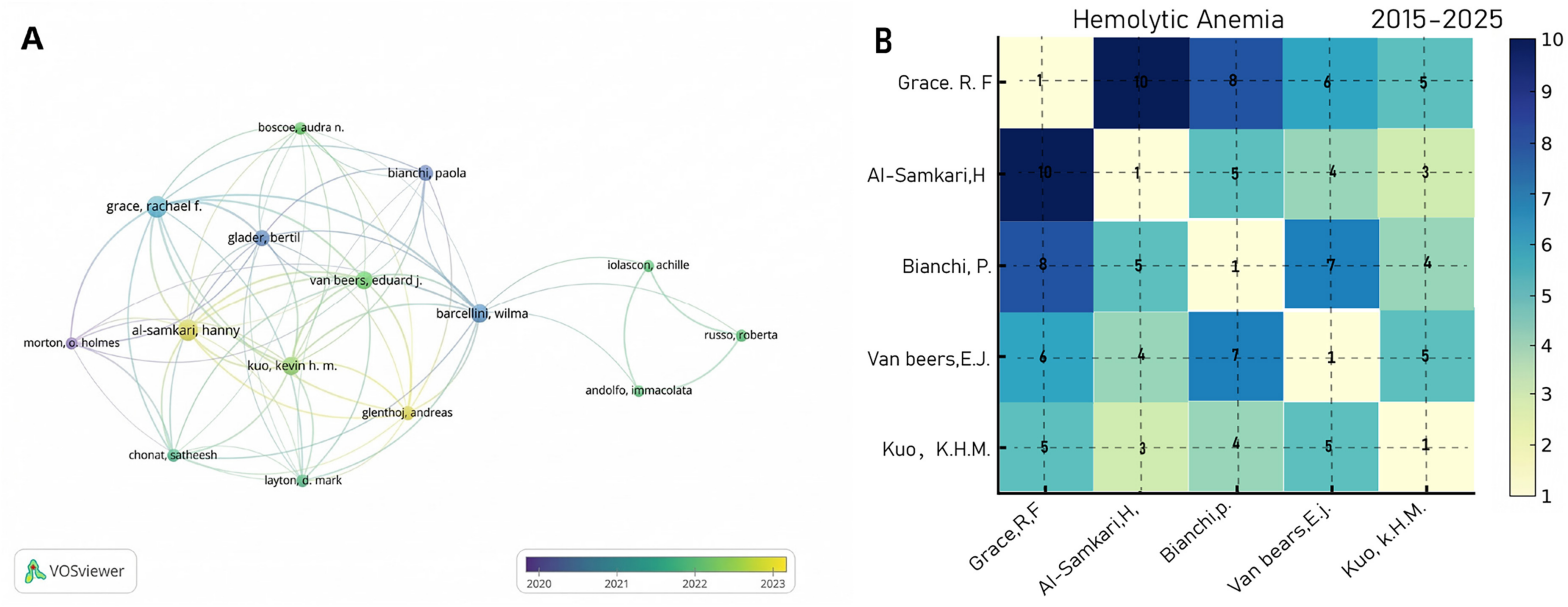
Author analysis. **(A)** Core author VOSviewer analysis chart. **(B)** Author collaboration heat map.

Grace, Rachael F. represents the most prolific independent contributor to the PKD landscape, leading the field with a publication count of 17 papers and a top-ranking co-citation frequency of 61 (Centrality = 0.08; Table 3), thereby exerting substantial intellectual weight. Concurrently, other core researchers like Barcellini, Wilma (8 publications, Centrality = 0.18) and van beers, Eduard J (7 publications, Centrality = 0.00) demonstrate strong academic impact. Notably, the collaborative axis between Barcellini and Van Beers anchors a significant transnational partnership, as visualized in Figure 5A. Broadly, the co-authorship network is defined by a multi-hub architecture, characterized by robust interdisciplinary integration and international synergy, as shown in the author collaboration matrix in Figure 5B.

**TABLE 3:** Top 10 most prolific authors and co-cited authors in PKD research.

Rank	Prolific author	Publication count (n)	Centrality	Co-cited author	Co-citation frequency (TC)	Centrality
1	Grace, Rachael F	17	0.08	Grace, Rachael F	61	0.09
2	Al-Samkari, Hanny	13	0.00	Alberto Zanella	51	0.09
3	Barcellini, Wilma	8	0.18	Ernest Beutler	44	0.26
4	Bianchi, Paola	8	0.00	Bianchi, Paola	36	0.03
5	van beers, Eduard J	7	0.00	UNKNOWN -	28	0.15
6	Chonat, Satheesh	6	0.00	Al-samkari, Hanny	27	0.12
7	Glader, Bertil	6	0.02	van beers, Eduard J	26	0.02
8	Kuo, Kevin H M	5	0.01	Charles Kung	20	0.09
9	Glenthoj, Andreas	5	0.04	Peter J. Carey	16	0.02
10	Fermo, Elisa	4	0.00	Achille Iolascon	13	0.29

**FIGURE 6:**
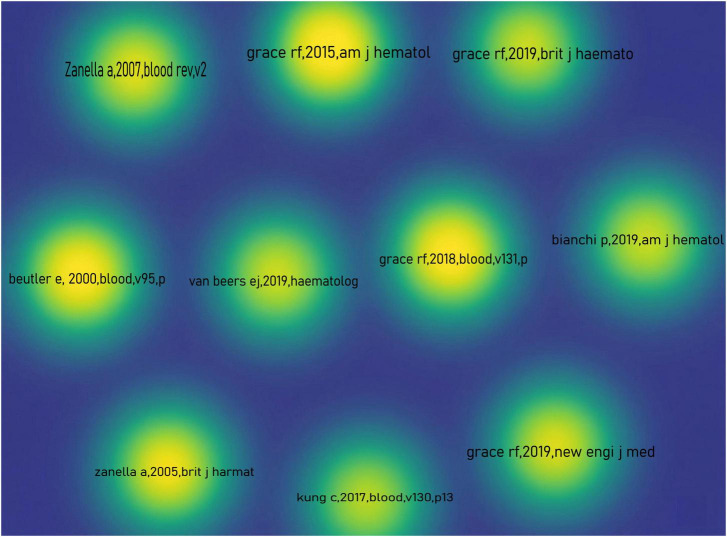
VOSviewer analysis of cited references.

A robust reference co-citation network has coalesced around central hematology literature, with Blood serving as a primary locus of structural inquiry in the field. This structural analysis confirms that the intellectual substrate of PKD research is situated at the intersection of hematology, molecular genetics, and clinical therapeutics, as shown in Figure 6.

#### Temporal evolution and keyword citation burst dynamics

**FIGURE 7:**
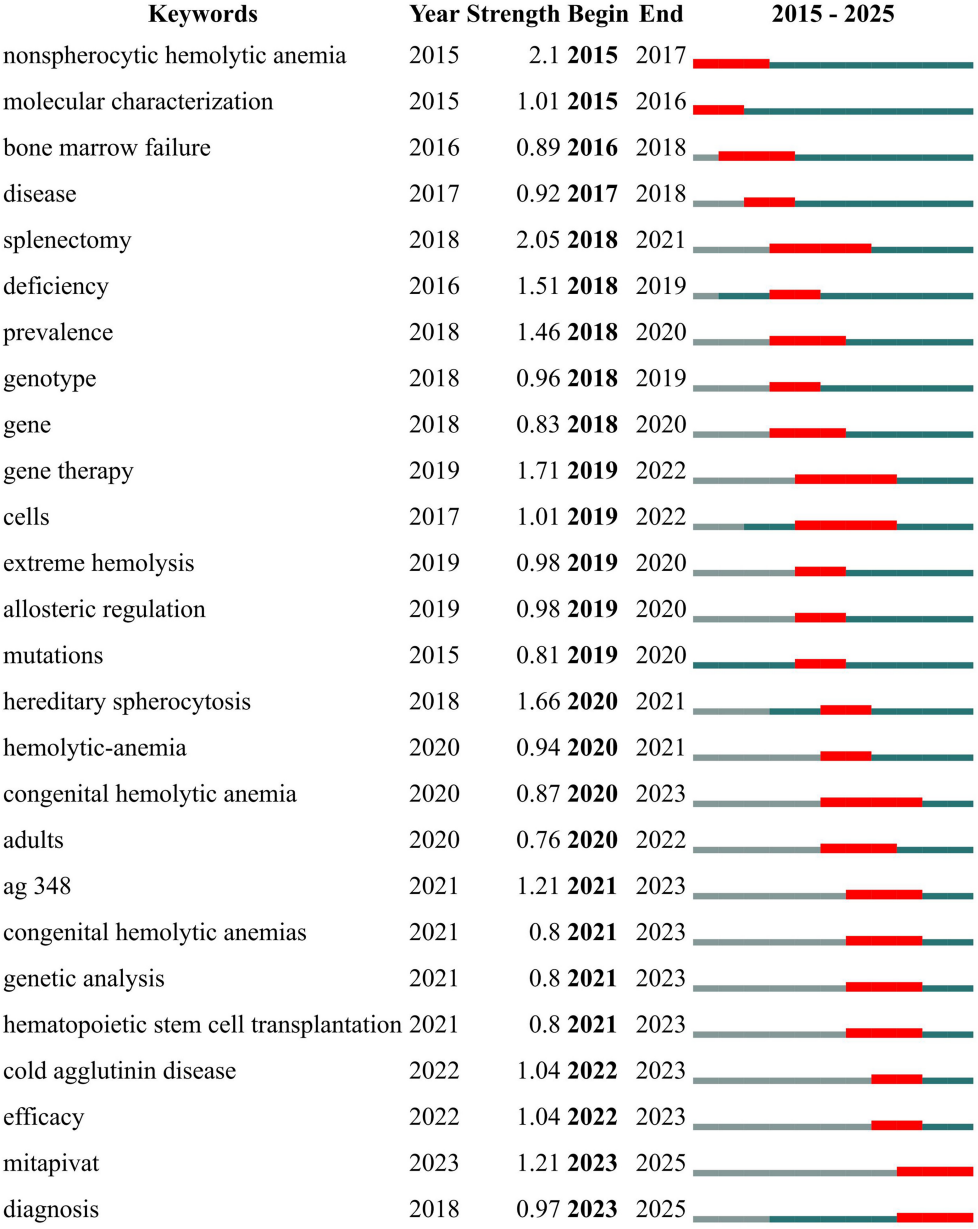
Top 26 keywords with the strongest citation bursts.

To delineate the temporal evolution of research priorities in the PKD domain, citation burst detection was performed on co-occurring keywords using Kleinberg’s algorithm. As shown in Figure 7, the top 26 burst keywords reveal a progressive shift in research emphasis over the past decade, which can be categorized into three chronological phases.

To mathematically evaluate the network’s structural reliability, topological validation metrics were computed. The Modularity Q score was 0.6589 (significantly exceeding the 0.3 threshold), demonstrating a robust and well-structured community division. The Mean Silhouette score was 0.8792 (well above the 0.7 threshold), indicating excellent cluster homogeneity and a highly reliable visual clustering configuration.

As detailed in Table 4, the keyword co-occurrence network was distilled into four predominant thematic clusters. Cluster #0 representing baseline clinical genetics and epidemiology; Cluster #3 focusing on conventional management support (e.g., iron overload and splenectomy); while Cluster #1 (Mitapivat/Activators, Mean Year: 2022.8) and Cluster #2 (Gene Therapy, Mean Year: 2020.5) chronologically capture the advanced therapeutic frontiers in PKD research over the decade.

**TABLE 4:** Top 15 high-frequency keywords and topological centrality in PKD research.

Cluster ID	Size (keywords)	Representative high-frequency keywords (top frequency)	Mean year
#0	23	Pyruvate kinase deficiency (50), hemolytic anemia (23), prevalence (17), mutations (13), diagnosis (12)	2017.2
#1	18	Mitapivat (4), safety (3), pyruvate kinase activator (2), agios pharmaceuticals (2), efficacy (2)	2022.8
#2	15	Gene therapy (4), lentiviral vector (2), clinical trial (2), hematopoietic stem cells (2), expression (2)	2020.5

The early stage (2015–2018) was primarily centered on clinical characterization and complication management, as reflected by prominent bursts in nonspherocytic hemolytic anemia (strength = 2.1, 2015–2017), bone marrow failure (strength = 0.89, 2016–2018), and splenectomy (strength = 2.05, 2018–2021). These terms demonstrate a core focus on disease phenotyping and conventional management.

The intermediate phase (2018–2022) indicates a growing emphasis on genetic mapping and epidemiological evaluation, supported by bursts in deficiency (strength = 1.51, 2018–2019), prevalence (strength = 1.46, 2018–2020), and gene therapy (strength = 1.71, 2019–2022). This period reflects an increasing interest in underlying molecular mechanisms and gene-based interventions.

In the most recent stage (2021–2025), research activity exhibits a clear transition toward targeted therapeutic development and clinical trial validation. This trend is highlighted by burst keywords such as AG-348 (strength = 1.21, 2021–2023), efficacy (strength = 1.04, 2022–2023), mitapivat (strength = 1.21, 2023–2025), and diagnosis (strength = 0.97, 2023–2025). These emerging topics track a definitive transition toward drug-oriented clinical evaluation.

Overall, the data indicate a gradual evolution from clinical description to genetic investigation, and more recently to therapy-oriented research, representing a continuous thematic transition rather than a sudden or discrete paradigm shift.

#### Exploratory cross-database thematic alignment analysis

A granular retrospective review of the 15 supplementary PubMed records (Supplementary Table S2) confirmed a stable thematic concordance with the evolutionary trajectories mapped by the primary WoSCC network. Quantitatively, 13.3% (*n* = 2, No. 5 and No. 15) of the supplementary validation corpus explicitly encapsulates the advanced therapeutic tier within the PKD domain. Specifically, this includes early-stage *ex vivo* lentiviral gene-editing optimization and localized clinical protocol frameworks for Phase II clinical trials evaluating the allosteric PKR activator mitapivat. Conversely, the remaining 86.7% (*n* = 13) of the records occupy the baseline clinical and diagnostic landscapes. These articles explore molecular heterogeneities of *PKLR* gene mutations across pediatric and international cohorts (No. 11, No. 13), novel real-time PCR genotyping methodologies (No. 3), fatal clinical complications such as post-splenectomy septic shock (No. 8), and critical patient-reported outcome (PRO) diary psychometric validations (No. 14). This precise distribution of sub-concepts mirrors the thematic transition tracked in our core dataset—solidifying the transition from baseline mechanistic mutation mapping to patient-centric clinical trial protocols and targeted therapeutics. Consequently, this qualitative intersection verifies that the primary WoSCC dataset securely captures the dominant boundaries of the discipline, confirming that the risk of missing critical scholarly hot spots due to single-database indexing is negligible.

## Discussion

### General bibliometric patterns and collaborative networks

By synthesizing a decade of literature (2015–2025) via the WoSCC, this analysis captures the evolving macro-level trajectory of PKD research. The empirical parameters demonstrate a structured topology where the United States represents the highest volume of scholarly output within the PKD landscape, anchoring a wide international network of institutional and sovereign collaborations. A defining feature of this global topology is the prominent integration of industrial stakeholders—most notably Agios Pharmaceuticals (Count = 17, Centrality = 0.12)—whose active participation underscores the pivotal role of cross-sector collaborations in catalyzing rare disease drug development.

On an individual and platform scale, prolific investigators, including Rachael F. Grace (Count = 17, Co-citation = 61) and Wilma Barcellini (Count = 8, Centrality = 0.18), function as central hubs in international networks. Moreover, co-citation mapping elucidates that the central publication nodes of PKD research are predominantly stratified within top-tier hematology journals, most notably Blood, Haematologica, and the British Journal of Haematology. This dense structural concentration signifies that the PKD knowledge base has crystallized at the critical confluence of hematology, erythrocyte metabolic pathology, and rare disease therapeutics.

### Clinical hotspots evolution and targeted therapeutic frontiers

While the field maintained a consistent growth pattern initially, the year 2020 represents a structural threshold, after which research contributions surged—reflecting a heightened clinical and scientific focus on the disease. This upward trajectory is directly tied to developments in clinical therapeutics. As a rare hereditary hemolytic anemia, PKD management was long constrained by a reliance on chronic transfusion support and splenectomy—a period marked by relatively stable research momentum.

However, the advent of mitapivat, a first-in-class small-molecule pyruvate kinase activator (12), together with the growing body of clinical evidence supporting its use (13), has catalyzed a redirection of academic inquiry. By allosterically modulating mutant PKR enzymes, mitapivat helps restore red cell metabolic homeostasis and improves hemoglobin levels in patients with PKD. This therapeutic advance has not only stimulated growing scholarly attention, but also reflects an important thematic transition in PKD management, moving from predominantly supportive care toward mechanism-based disease-modifying interventions.

Accordingly, recent research hotspots have increasingly focused on mitapivat’s mechanism of action, long-term safety, and its implications for clinical practice. Prolific investigators’ research trajectories form the clinical evidence base for contemporary management recommendations and international expert guidance (14, 15). Keyword clustering and frontier detection delineate a clear thematic transition within the PKD research landscape. The canonical knowledge base was traditionally anchored in descriptive genotype-phenotype correlations (16), enzymatic assays, and the clinical sequelae of splenectomy (17).

In contrast, gene therapy should be interpreted as an emerging and exploratory frontier rather than a dominant current evidence base in PKD research. Although this direction has attracted growing conceptual interest, its bibliometric representation in the present study remains limited compared with that of PKR activators. Therefore, the current therapeutic landscape is more appropriately characterized as being primarily driven by the clinical development of mitapivat, while gene-based strategies remain at an earlier stage of investigation. In parallel, landmark clinical studies and registry-based investigations indicate that the field is increasingly moving toward longitudinal clinical evaluation and a broader assessment of disease burden, treatment outcomes, and patient-centered management.

Looking ahead, future PKD research is likely to continue along two broad directions. The first is the further clinical evaluation of PKR activators, particularly with respect to long-term efficacy, safety, durability of response, and broader applicability across diverse patient populations, including underrepresented groups such as non-transfusion-dependent individuals, pediatric patients, and pregnant patients (18, 19). In parallel, the development of next-generation PK activators may further improve pharmacokinetic properties and treatment accessibility, aligning with the broader expansion of therapeutic options across various rare anemia spectrums. The second is the continued exploration of gene-based strategies as an emerging investigational direction. Although such approaches hold conceptual promise, their current representation in the PKD literature remains limited, and further experimental and translational studies are needed before they can be considered a major therapeutic pillar.

In addition, future therapeutic research may increasingly consider combination-based and individualized management strategies. For example, PKR activators may be evaluated alongside supportive interventions such as iron chelation in order to better address chronic complications including refractory anemia and systemic iron overload (5, 18). However, such approaches remain largely prospective and should be interpreted cautiously until supported by more direct clinical evidence (20).

In summary, the PKD research landscape is evolving from foundational mechanistic investigation toward more clinically oriented and mechanism-informed therapeutic research. By applying systematic bibliometric analysis, this study maps the field’s shift from conventional supportive management toward targeted intervention and patient-centered clinical evaluation. These findings provide a structured overview of current research priorities and may help inform future clinical investigation, evidence synthesis, and strategic research planning.

## Data Availability

The original contributions presented in the study are included in the article/Supplementary material, further inquiries can be directed to the corresponding author.
