# Supplementary material for: Global research landscape and thematic transitions in pyruvate kinase deficiency: a decadal bibliometric analysis (2015–2025)
Source: Front Med (Lausanne). 2026 Jun 24;13:1865929. doi: 10.3389/fmed.2026.1865929 (PMC13342182; doi:10.3389/fmed.2026.1865929)
Supplement: Supplementary file 1 [file Data_Sheet_1.pdf]

Supplementary Table S1.

Detailed Database Search Strategies and Technical Syntax Framework.

Database / Repository	Search Field	Exact Search Syntax / Query	Technical Restrictions & Filters Applied	Records Retrieved	Screening & Eligibility Status
Web of Science Core Collection (WoSCC)	Topic (TS)	TS=("Pyruvate Kinase Deficiency")	• Indexes: SCI-EXPANDED, SSCI<br>• Time Span: July 2015 – July 2025<br>• Document Types: Article, Review<br>• Language: English	649	• 90 publications selected for primary network/visualized analysis after rigorous screening (62 articles, 28 reviews).<br>• Full bibliographic metadata exported.
PubMed	Title/Abstract	"Pyruvate Kinase Deficiency"[Title/Abstract]	• Time/Publication Date: July 2015 – July 2025<br>• Document Types: Journal Article, Review<br>• Language: English<br>• *Note: MeSH terms intentionally excluded.*	188	• 15 non-overlapping records identified.<br>• Retained exclusively for external thematic concordance checks.<br>• Excluded from co-occurrence networks due to metadata disparities.
Scopus	Title / Abstract / Keywords	TITLE-ABS-KEY("Pyruvate Kinase Deficiency")	• Time/Publication Date: July 2015 – July 2025<br>• Document Types: Article, Review<br>• Language: English	0	• 0 additional eligible records yielded beyond the primary WoSCC set.<br>• Logged for negative-control methodological validation.
