# Supplementary material for: Global research landscape and thematic transitions in pyruvate kinase deficiency: a decadal bibliometric analysis (2015–2025)
Source: Front Med (Lausanne). 2026 Jun 24;13:1865929. doi: 10.3389/fmed.2026.1865929 (PMC13342182; doi:10.3389/fmed.2026.1865929)
Supplement: Supplementary file 2 [file Data_Sheet_2.pdf]

Supplementary Table S2.

PubMed records (n=15) identified in external validation and not overlapping with WoSCC dataset, with topic tags (mitapivat/gene therapy).

Note: The topic tag (✓/—) indicates whether each record explicitly involves mitapivat or gene/gene-editing therapy concepts, based on titles and citation strings. These records were used for qualitative thematic concordance and were not merged into the primary WoSCC dataset due to minimal sample size and incomplete bibliometric metadata for stable network analyses.

No.	PMID	DOI	Year	Title	Publication Type	Journal/Citation	Mitapivat/Gene therapy topic
**1**	25388786	10.1002/ajh.23899	2015	Partial pyruvate kinase deficiency aggravates the phenotypic expression of band 3	Journal Article	Am J Hematol. 2015 Mar;90(3):E35-9. doi: 10.1002/ajh.23899. Epub 2014 Nov 24.	—
**2**	25482511	10.1016/j.molcel.2014.10.027	2015	Pyruvate kinase isoform expression alters nucleotide synthesis to impact cell	Journal Article; Research Support, N.I.H., Extramural; Research Support, Non-U.S. Gov't; Research Support, U.S. Gov't, Non-P.H.S.	Mol Cell. 2015 Jan 8;57(1):95-107. doi: 10.1016/j.molcel.2014.10.027. Epub 2014	—
**3**	25716288	10.1292/jvms.14-0600	2015	Real-time PCR genotyping assay for feline erythrocyte pyruvate kinase deficiency	Journal Article; Research Support, N.I.H., Extramural; Research Support, Non-U.S. Gov't	J Vet Med Sci. 2015 Jun;77(6):743-6. doi: 10.1292/jvms.14-0600. Epub 2015 Feb 13.	—
**4**	26300261	10.1016/j.molcel.2015.07.013	2015	Lack of Evidence for PKM2 Protein Kinase Activity.	Journal Article; Research Support, N.I.H., Extramural; Research Support, Non-U.S. Gov't	Mol Cell. 2015 Sep 3;59(5):850-7. doi: 10.1016/j.molcel.2015.07.013. Epub 2015	—
**5**	26549847	10.1016/j.stemcr.2015.10.002	2015	Generation of a High Number of Healthy Erythroid Cells from Gene-Edited Pyruvate	Journal Article; Research Support, Non-U.S. Gov't	Stem Cell Reports. 2015 Dec 8;5(6):1053-1066. doi: 10.1016/j.stemcr.2015.10.002.	✓
**6**	26733775	10.1159/000438776	2015	The Diagnostic Utility of Single Long Contiguous Stretches of Homozygosity in	Journal Article	Mol Syndromol. 2015 Sep;6(3):135-40. doi: 10.1159/000438776. Epub 2015 Aug 15.	—
**7**	26903544	10.1182/blood-2016-01-694331	2016	Krüppeling erythropoiesis: an unexpected broad spectrum of human red blood cell	Journal Article; Research Support, N.I.H., Extramural; Research Support, Non-U.S. Gov't; Review	Blood. 2016 Apr 14;127(15):1856-62. doi: 10.1182/blood-2016-01-694331. Epub 2016	—
**8**	27610253	10.1155/2016/1538501	2016	Plesiomonas shigelloides Septic Shock Leading to Death of Postsplenectomy Patient	Journal Article	Case Rep Infect Dis. 2016;2016:1538501. doi: 10.1155/2016/1538501. Epub 2016 Aug	—
**9**	28542307	10.1371/journal.pone.0177818	2017	The mouse Char10 locus regulates severity of pyruvate kinase deficiency and	Journal Article	PLoS One. 2017 May 18;12(5):e0177818. doi: 10.1371/journal.pone.0177818.	—
**10**	29445082	10.1038/s41419-018-0296-4	2018	Pyruvate kinase M2 regulates photoreceptor structure, function, and viability.	Journal Article; Research Support, N.I.H., Extramural; Research Support, Non-U.S. Gov't	Cell Death Dis. 2018 Feb 14;9(2):240. doi: 10.1038/s41419-018-0296-4.	—
**11**	29519373	10.1016/j.bjhh.2017.08.007	2018	Novel mutations associated with pyruvate kinase deficiency in Brazil.	Journal Article	Hematol Transfus Cell Ther. 2018 Jan-Mar;40(1):5-11. doi:	—
**12**	33551834	10.3389/fphys.2020.609103	2020	A Proposed Concept for Defective Mitophagy Leading to Late Stage Ineffective	Journal Article	Front Physiol. 2021 Jan 20;11:609103. doi: 10.3389/fphys.2020.609103. eCollection	—
**13**	37466302	10.1002/mgg3.2239	2023	Clinical outcome and genotype analysis of four Chinese children with pyruvate	Journal Article	Mol Genet Genomic Med. 2023 Nov;11(11):e2239. doi: 10.1002/mgg3.2239. Epub 2023	—
**14**	37943362	10.1186/s41687-023-00650-3	2023	Psychometric validation of the Pyruvate Kinase Deficiency Diary and Pyruvate	Clinical Trial, Phase III; Journal Article; Research Support, Non-U.S. Gov't	J Patient Rep Outcomes. 2023 Nov 9;7(1):112. doi: 10.1186/s41687-023-00650-3.	—
**15**	39079928	10.1136/bmjopen-2023-083691	2024	Designing a single-arm phase 2 clinical trial of mitapivat for adult patients	Clinical Trial Protocol; Journal Article; Research Support, Non-U.S. Gov't	BMJ Open. 2024 Jul 30;14(7):e083691. doi: 10.1136/bmjopen-2023-083691.	✓
